# Do topical repellents divert mosquitoes within a community?

**DOI:** 10.1186/1475-2875-11-S1-P120

**Published:** 2012-10-15

**Authors:** Marta Maia, Peter Sangoro, Max Thele, Elizabeth Turner, Sarah Moore

**Affiliations:** 1 Department of Disease Control, London School of Hygiene & Tropical Medicine, London, WC1E 7HT, UK; 2 Environmental Group, Ifakara Health Institute, Bagamoyo, United Republic of Tanzania; 3 Institut Franco-Allemand de Recherche de Saint Louis, St. Louis, France; 4 Departments of Biostatistics and Bioinformatics, Duke Global Health Institute, NC, USA

## Background

Repellents are compounds which interfere with the mosquito’s olfactory system hindering them to identify their hosts and succeeding in taking a blood-meal [1]. However, repellents do not eliminate the host-seeking mosquitoes, they simply reduce human-vector contact. Consequently, there is a possibility that individuals, who do not use repellents, experience more bites than usual because mosquitoes are diverted from the repellent users. The objective of this study was to measure if diversion occurs from households that use repellents to those that don’t within a community with incomplete topical repellent coverage.

## Materials and methods

An interventional study was performed in three villages of southern Tanzania using 15%-DEET (N,N-Diethyl-meta-toluamide) and a placebo lotion. Three coverage scenarios were investigated: complete repellent coverage (all households were given 15%-DEET), incomplete repellent coverage (80% of households were given DEET-15% and 20% were given a placebo lotion) and no repellent coverage (all households were given a placebo lotion). The coverage scenarios were rotated between villages. Mosquito densities were measured through aspiration of indoor and outdoor resting mosquitoes respective to each enrolled household. Data was analysed using mixed-effects models and the no coverage scenario was used as reference.

## Results

Placebo users living in a village where 80% of the households used 15%-DEET were likely to have nearly three times more mosquitoes (p<0.001) resting in their dwellings in comparison to households in a village where nobody uses repellent (Table 1).

**Table 1:** Incidence rate ratios (IRR), model estimated means^1^, confidence intervals and p-values of mean number of mosquitoes aspirated per night per household by treatment group

	IRR	Mean^1^	95% CI	p-value
No coverage	1	4.97	[3.77 - 6.16]	-
Complete coverage	0.5	2.49	[1.76 - 3.22]	<0.001
80% Coverage (repellent users)	0.69	3.45	[2.83 - 4.06]	0.015
80% Coverage (non repellent users)	2.87	14.25	[9.74 - 18.76]	<0.001

## Conclusions

There is strong evidence that mosquitoes are diverted between households that use repellent to those that don’t. This study arises questions on health equity associated with repellent usage. Policy makers should take into consideration these results while devising vector control programs, as less privileged individuals are likely to suffer more mosquito bites and therewith be more exposed to vector-borne diseases if universal coverage is not reached.
